# Stacked deep analytic model for human activity recognition on a UCI HAR database

**DOI:** 10.12688/f1000research.73174.3

**Published:** 2022-04-01

**Authors:** Ying Han Pang, Liew Yee Ping, Goh Fan Ling, Ooi Shih Yin, Khoh Wee How

**Affiliations:** 1 Faculty of Information Science and Technology, Multimedia University, Ayer Keroh, Melaka, 75450, Malaysia; 2 Millapp Sdn Bhd, Bangsar South, Kuala Lumpur, 59200, Malaysia

**Keywords:** smartphone, one-dimensional motion signal, activity recognition, stacking deep network, discriminant learning

## Abstract

Background

Owing to low cost and ubiquity, human activity recognition using smartphones is emerging as a trendy mobile application in diverse appliances such as assisted living, healthcare monitoring, etc. Analysing this one-dimensional time-series signal is rather challenging due to its spatial and temporal variances. Numerous deep neural networks (DNNs) are conducted to unveil deep features of complex real-world data. However, the drawback of DNNs is the un-interpretation of the network's internal logic to achieve the output. Furthermore, a huge training sample size (i.e. millions of samples) is required to ensure great performance.

Methods

In this work, a simpler yet effective stacked deep network, known as Stacked Discriminant Feature Learning (SDFL), is proposed to analyse inertial motion data for activity recognition. Contrary to DNNs, this deep model extracts rich features without the prerequisite of a gigantic training sample set and tenuous hyper-parameter tuning. SDFL is a stacking deep network with multiple learning modules, appearing in a serialized layout for multi-level feature learning from shallow to deeper features. In each learning module, Rayleigh coefficient optimized learning is accomplished to extort discriminant features. A subject-independent protocol is implemented where the system model (trained by data from a group of users) is used to recognize data from another group of users.

Results

Empirical results demonstrate that SDFL surpasses state-of-the-art methods, including DNNs like Convolutional Neural Network, Deep Belief Network, etc., with ~97% accuracy from the UCI HAR database with thousands of training samples. Additionally, the model training time of SDFL is merely a few minutes, compared with DNNs, which require hours for model training.

Conclusions

The supremacy of SDFL is corroborated in analysing motion data for human activity recognition requiring no GPU but only a CPU with a fast- learning rate.

## Introduction

Human activity recognition (HAR) can be categorized into vision-based and sensor-based. In vision-based HAR, an image sequence, in the form of video, recording the human activity is captured by a camera. ^1^ This sequence will be analysed to recognize the nature of an action. This system is applied for surveillance, human-computer interaction and healthcare monitoring. For sensor-based HAR, human activities are captured by inertial sensors, such as accelerometers, gyroscopes or magnetometers. Among these approaches, sensors are more favourable due to their lightweight nature, portability and low energy usage. ^2^ With the advancement of mobile technology, smartphones are equipped with high-end components. Accelerometer and gyroscope sensors embedded in the smartphone make it feasible as an acquisition device for HAR. Smartphone-based HAR has been an area of contemporary research in recent years. ^3– 6^ In this work, we categorize the smartphone-based HAR as part of the sensor-based HAR. Activity inertial signals are collected through smartphone sensors.

### Related work

Hand-crafted approaches using manually computed statistical features have been proposed. ^4, 7^ These authors applied various machine learning techniques such as decision tree, logistic regression, multilayer perceptron, naïve Bayes, Support Vector Machine etc. to classify the detected activities. The performance of the handcrafted approaches might be affected when dealing with complex scenarios due to their feature representation incapability. The algorithms could easily plummet into the local minimum despite the global optimal.

Hence, various deep neural networks (DNNs) were explored in HAR owing to the capability of extracting informative features. DNN is a machine learner that can automatically unearth the data characteristics hierarchically from lower to deeper levels. ^8^^,^^9^ The work of Ronao and Cho (2016), ^10^ Lee *et al*. (2017) ^11^ and Ignatov (2018) ^12^ explored the deep convolutional neural networks by exploiting the activity characteristics in the one-dimensional time-series signals captured by the smartphone inertial sensors. The empirical results substantiated that the extracted deep features were crucial for data representation with promising recognition performance.

Zeng *et al*. (2014) proposed a modified convolutional neural network to extract scale-invariant characteristics and local dependency of the acceleration time-series signal. ^13^ The weight sharing mechanism in the convolutional layer was modified. Unlike in the vanilla model where the local filter weights were shared by all positions within the input space, the authors incorporated a more relax weight sharing strategy (partial weight sharing) to enhance the performance.

Recurrent Neural Network (RNN) was proposed to process sequential data by analysing previously inputted data and processing it linearly. Due to the vanishing gradient problem, RNN was enhanced and Long Short-Term Memory – LSTM was introduced. Chen *et al*. (2016) explored the feasibility of LSTM in predicting human activities. ^14^ Empirical results demonstrated an encouraging performance of LSTM in HAR. Further, an enhanced version of LSTM, i.e. bidirectional LSTM, was proposed. ^15^ Unlike LSTM, bidirectional LSTM tackles both past and future information during the feature analysis. With this, a richer description of features could be extracted for classification.

A cascade ensemble learning (CELearning) model was proposed for smartphone-based HAR. ^16^ There are multiple layers in this aggregation network and the model goes deeper layer by layer. Each layer contains Extremely Gradient Boosting Trees, Random Forest, Extremely Randomized Trees and Softmax Regression. The CELearning model gains higher performance, and the training process is rather simple and efficient. Besides, Hierarchical Multi-View Aggregation Network (HMVAN) is also one of the aggregation models. ^17^ This model integrates features from various feature spaces in a hierarchical context. In this network, three aggregation modules from the aspect of feature, position and modality levels are designed. Further, a modified Dynamic Time Warping (DTW) has been proposed for template selection in human activity recognition. ^18^ Empirical results showed that the modified DTW was able to improve on the computational efficiency and similarity measure accuracy. In viewing the significance of time-series and continuous characteristics of sensor data, a two-stage continuous hidden Markov model framework was proposed by taking advantage of the innate hierarchical structure of basic activities. ^19^ This framework could diminish feature computation overhead by manipulating different feature subsets on different subclasses. Experiments showed that this proposed hierarchical structure drastically boosted the recognition performance.

### Motivation and contributions

In DNNs, there are learning modular components in multiple processing layers for multiple-level feature abstraction. These layers are trained based on a versatile learning principle, no requiring any manual design by experts. ^20^ DNNs accomplish excellent performances. However, these networks are not well trained if they have limited training samples, leading to performance degradation. Furthermore, there is a lack of theoretical ground on how to fine-tune the gigantic hyper-parameter series. ^17^ The outstanding accomplishment of DNNs can only be achieved if and only if sufficient training data is accessible for fine-tuning the numerous parameters. A high specification of GPU is needed to train the network from gargantuan datasets. Besides, impersonal HAR solution is preferable for real-time applications. This solution can be directly applied to new users without necessitating model regeneration.

A stacking-based deep learning model for smartphone-based HAR is proposed. Inspired by the hierarchical learning in the DNNs, the proposed stacked learning network is aggregated with multiple learning modules, one after another, in a hierarchical framework. Specifically, a discriminant learning function is implemented in each module for discriminant mapping to generate discriminative features, level by level. The lower (generic) to deeper features are input to a classifier for activity identification. This proposed approach is termed Stacked Discriminant Feature Learning (SDLF).

The contributions of this work are summarized:

1. A deep analytic model is proposed for smartphone based HAR for quality feature extraction without the need of a gigantic training set and tenuous hyper-parameter tuning.
2. An adaptable modular model is developed with a discriminant learning function in each module to extract discriminant deep features demanding no graphics processing unit (GPU) but only a central processing unit (CPU) with a fast-learning rate.
3. An experimental analysis using various performance evaluation metrics (i.e. recall, precision, the area under the curve, computational time, etc.) with subject-independent protocol implementation (no overlap in subjects between training and testing sets) to facilitate impersonal HAR solution.

## Methods

**Figure 1.**
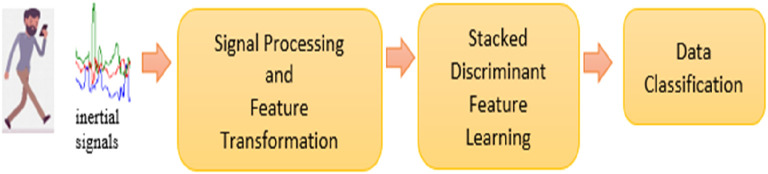
Overview of the proposed Stacked Discriminant Feature Learning (SDFL) system.

Smartphone inertial sensors were used to capture 3-axial linear (total) acceleration and 3-axial angular velocity signals. These signals were pre-processed into time- and frequency-domain features, as listed in Table 1. Next, the pre-processed data was inputted into the Stacked Discriminant Feature Learning (SDFL) for feature learning. The extracted feature template was fed into the nearest-neighbour (NN) classifier for classification. The overview of the system is illustrated in Figure 1.

**Table 1.** Pre-processed features as input data into the Stacked Discriminant Feature Learning (SDFL) system.

Function	Feature
Mean	Average value
Std dev	Standard deviation
Median	Median absolute value
Max	Largest value in array
Min	Smallest value in array
Sma	Signal magnitude area
Energy	Average sum of squares
Iqr	Interquartile range
Entropy	Signal entropy
ArCoeff	Auto-regression coefficients
Correlation	Correlation coefficient
MaxFreqInd	Largest frequency component
MeanFreq	Frequency signal weighted average
Skewness	Frequency signal skewness
Kurtosis	Frequency signal kurtosis
EnergyBand	Energy of a frequency interval
Angle	Angle between two vectors

**Figure 2.**
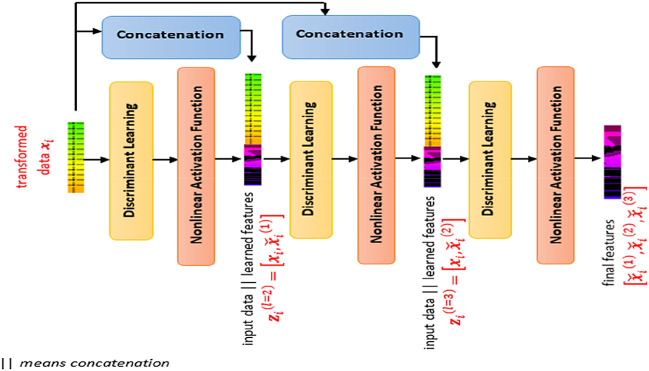
Stacked Discriminant Feature Learning (SDFL) framework.

SDFL is a pile of multiple discriminant learning layers interleaved with a nonlinear activation unit, as illustrated in Figure 2. By cascading multiple discriminant learning modules, each layer of SDFL learns based on the input data and the learned nonlinear features of the preceding module. The depth of the stacking layer is determined using the database subset. If the performance is not improving but showing degradation, the depth of the stacking layer is determined. In this case, the depth of three showed the optimal performance, so we adopted this architecture with three layers. To be detailed, the first discriminant learning module learns based on the input data and the second learning module learns based on an input vector (concatenating the input data and the learned features of the first learning module). This is similar to the third learning process where the third learning module learns based on an input vector (comprising the input data and the learned features of the second learning module).

Let ${\left\{\left(x_{i},y_{i}\right)\right\}}_{i=1}^{N}$ be a set of N transformed data with *d* dimension, i.e. *d* = 561, $y_{i}$ is the class label of $x_{i}$ , C is the number of training classes, i.e. *C* = 6, each of C classes has a mean $\mu_{j}$ and total mean vector $\mu =\frac{1}{C}\sum_{j=1}^{C}m_{j}\mu_{j}$ with $m_{j}$ denotes the number of training samples for *j*th class. In the first learning layer, the input vector is the transformed data $x_{i}$ . The computation of the intrapersonal scatter matrix $\Sigma_{\text{intra}}$ and interpersonal scatter matrix $\Sigma_{\text{inter}}$ are defined as:

$$\Sigma_{\text{intra}}=\sum_{j=1}^{C}\sum_{x_{i},y_{i}=C_{j}}\left(x_{i}-\mu_{j}\right){\left(x_{i}-\mu_{j}\right)}^{T}$$ (1)

$$\Sigma_{\text{inter}}=\sum_{j=1}^{C}\mu_{j}\left(\mu_{j}-\mu \right){\left(\mu_{j}-\mu \right)}^{T}$$ (2)

where T denotes a transpose operation. Next, a linear transformation Φ is computed by maximizing the *Rayleigh coefficient*. With this optimization, the data from the same person could be projected close to each other, while data from different people is projected as far apart as possible. This optimization function is termed as Fisher’s criterion, ^21^

$$J\left(\Phi \right)=\frac{\left|\Phi^{T}\Sigma_{\text{inter}}\Phi \right|}{\left|\Phi^{T}\Sigma_{\text{intra}}\Phi \right|}$$ (3)

The mapping Φ is constructed through solving the generalized eigenvalue problem,

$$\Sigma_{\text{inter}}\Phi ={\lambda \Sigma }_{\text{intra}}\Phi$$ (4)

The learned features are produced through the projection of the input data $x_{i}$ onto the mapping subspace,

$${\hat{x}}_{i}=\Phi^{T}x_{i}$$ (5)

$\hat{x}$ is transformed to C−1 dimensions. We denote *l* for the index of modular layer in SDFL. The learned feature vector of the first modular unit is notated as ${{\hat{x}}_{i}}^{\left(l=1\right)}={{\hat{x}}_{i}}^{\left(1\right)}$ . A nonlinear input-output mapping is applied to ${{\hat{x}}_{i}}^{\left(1\right)}$ via a nonlinear activation function. In this study, sigmoid function, ${\overset{ˇ}{x}}_{i}=S\left({\hat{x}}_{i}\right)=\frac{1}{1+e^{-{\hat{x}}_{i}}}$ is used for the nonlinear projection. To be specific, ${{\overset{ˇ}{x}}_{i}}^{\left(1\right)}=\frac{1}{1+e^{-{{\hat{x}}_{i}}^{\left(1\right)}}}$ is the nonlinear learned features of the first modular unit.

For deeper modules, the input vector of the respective module is a stacking vector containing the input data and the learned features, i.e. ${z_{i}}^{\left(l\right)}=\left[x_{i},{{\overset{ˇ}{x}}_{i}}^{\left(l-1\right)}\;\right]$ where l=2 and 3 . The intrapersonal scatter matrix ${\Sigma_{\text{intra}}}^{\left(l\right)}$ and interpersonal scatter matrix ${\Sigma_{\text{inter}}}^{\left(l\right)}$ are formulated,

$${\Sigma_{\text{intra}}}^{\left(l\right)}=\sum_{j=1}^{C}\sum_{{z_{i}}^{\left(l\right)}\in C_{j}}\left({z_{i}}^{\left(l\right)}-{\mu_{j}}^{\left(l\right)}\right){\left({z_{i}}^{\left(l\right)}-{\mu_{j}}^{\left(l\right)}\right)}^{T}$$ (6)

$${\Sigma_{\text{inter}}}^{\left(l\right)}=\sum_{j=1}^{C}{\mu_{j}}^{\left(l\right)}\left({\mu_{j}}^{\left(l\right)}-\mu^{\left(l\right)}\right){\left({\mu_{j}}^{\left(l\right)}-\mu^{\left(l\right)}\right)}^{T}$$ (7)

In this case, ${\mu_{j}}^{\left(l\right)}$ is the *j*th class mean computed from the input vectors of *j*th class, ${z_{i}}^{\left(l\right)}\in C_{j}$ and the total mean vector $\mu^{\left(l\right)}=\frac{1}{N}\sum_{i=1}^{N}m_{j}{\mu_{j}}^{\left(l\right)}$ at *l*th modular unit. The final feature vector is the nonlinear learned features of each modular layer with length of 3( *C*-1),

$${{\overset{ˇ}{x}}_{i}}^{\text{final}}=\left[{{\overset{ˇ}{x}}_{i}}^{\left(1\right)},{{\overset{ˇ}{x}}_{i}}^{\left(2\right)},{{\overset{ˇ}{x}}_{i}}^{\left(3\right)}\right]$$ (8)

In this work, the experimental hardware platform was constructed on a desktop with an Intel ^®^ Core™ i7-7700 processor with 4.20 GHz and 48.0 GB main memory; whereas the experimental software platform was a 64-bit operating system of Windows 10 with Matlab R2018a (MATLAB, RRID:SCR_001622) software (An open-access alternative that provides an equivalent function is GNU Octave (GNU Octave, RRID:SCR_014398)). We used the UCI HAR dataset ^7^: There were 30 subjects with 7352 training samples and 2947 testing samples. Each subject was required to carry a smartphone (Samsung Galaxy SII) on the waist and perform six activities: walking, walking_upstairs, walking_downstairs, sitting, standing and laying.

## Results

**Figure 3.**
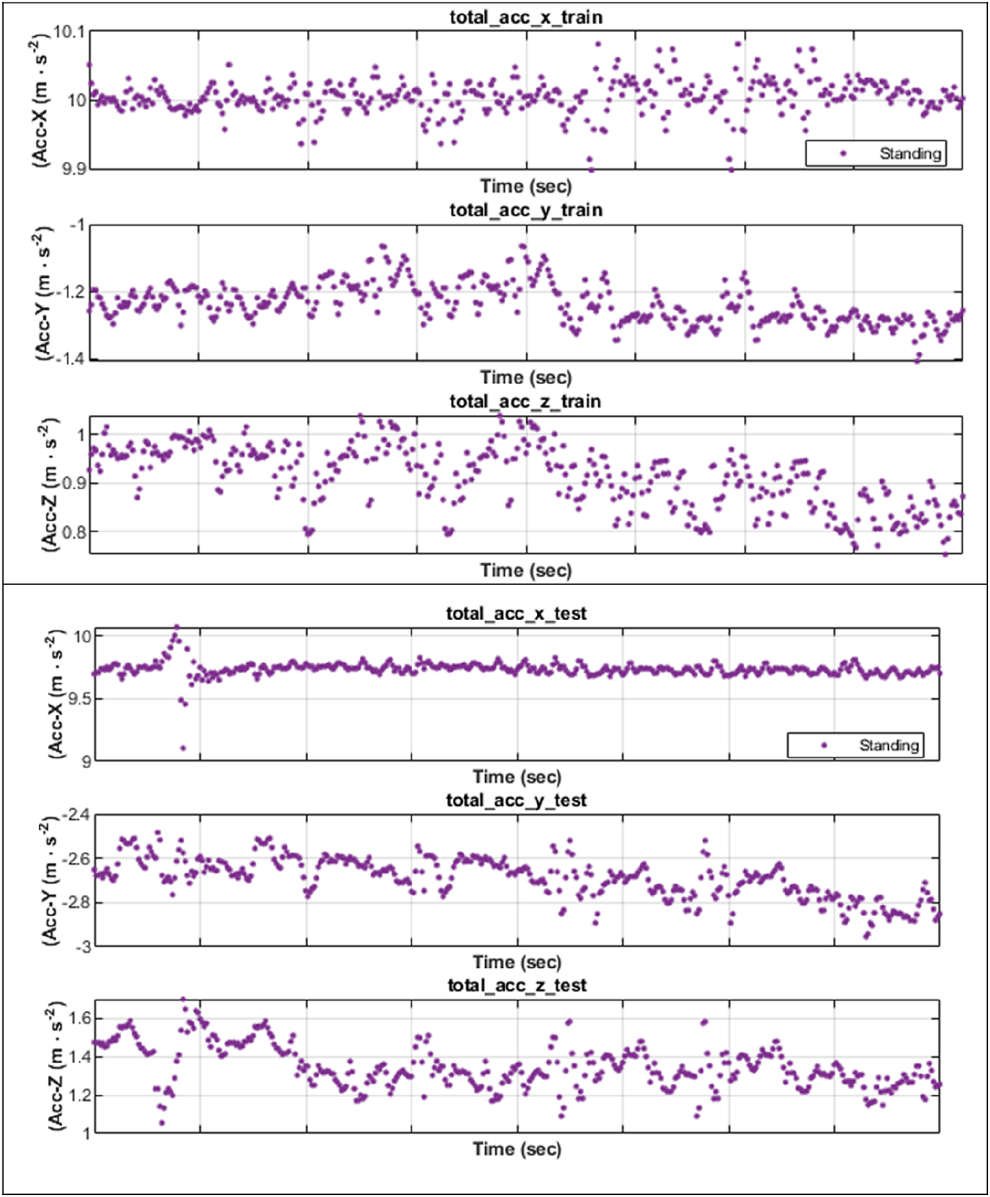
Different inertial data patterns of standing between two subjects.

We scrutinized how well SDFL could analyse the inertial data and correctly classify those activities in a user-independent scenario. The challenge of subject-independent protocol is that there is a certain degree of variance of human gait towards the inertial data patterns, although performing the same activity, as illustrated in Figure 3 (for standing).

**Figure 4.**
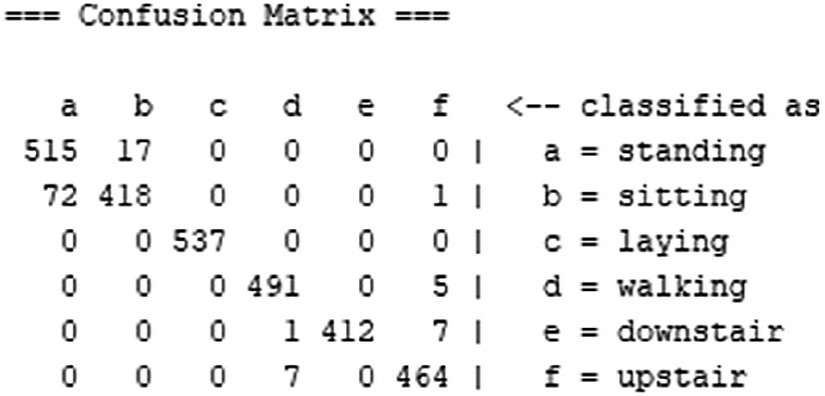
Confusion matrix of Stacked Discriminant Feature Learning (SDFL).

In this work, UCI HAR dataset was partitioned into two sets: 70% of the volunteers were selected to generate the training data and the remaining 30% of the volunteers’ data was used as the testing data. There was no subject overlapping between the training and test data sets. Table 2 records the performance of SDFL and Figure 4 shows the confusion matrix. The performance comparison with other approaches is recorded in Table 3. The computational time of SDFL is tabulated in Table 4.

**Table 2.** Performance of Stacked Discriminant Feature Learning (SDFL).

Metric	Performance
True Positive (TP) rate	0.963
False Positive (FP) rate	0.008
Precision	0.964
Recall	0.963
F-score	0.963
Area Under the Curve	0.977
Accuracy (%)	96.2674

**Table 3.** Performance comparison of Stacked Discriminant Feature Learning (SDFL) with alternative approaches.

Method	Accuracy (%)
Multiclass Support Vector Machine ^7^	96
Dynamic Time Warping ^18^	89.00
Hierarchical Continuous Hidden Markov Model ^19^	93.18
Deep Belief Network (as reported in ^9^)	95.80
Group-based Context-aware method for human activity recognition (GCHAR) ^3^	94.16
Handcrafted Cascade Ensemble Learning model (CELearning) ^16^	96.88
Automated Cascade Ensemble Learning model (CELearning) ^16^	95.93
Convolutional Neural Network (CNN) ^10^	95.75
Artificial Neural Network (ANN) (as reported in ^10^)	91.08
Stacked Discriminant Feature Learning (SDFL)	96.27

**Table 4.** Computational time of Stacked Discriminant Feature Learning (SDFL). Classifier = Nearest Neighbour (NN) classifier.

System phase		Computational time (s)
Training (7352 instances)	Model training	0.468258
	Classification	2.67
	**Total training**	**3.138258**
Testing (2947 instances)	Data learning	0.017416
	Classification	1.22
	**Total testing**	**1.237416**

## Discussion

From the empirical results, we observed that the proposed SDFL was able to demonstrate superior classification performance compared to most of the existing techniques, even though a simple classifier was adopted in the system. The exceptional performance of SDFL explains the capability of SDFL in capturing the essence of the inertial data without heavily depending on the classifier. Furthermore, SDFL also exhibited its superiority to most of the existing approaches, including deep learning models. To be specific, SDFL obtained an accuracy of 96.3%, whilst Deep Belief Network’s accuracy was 95.8%, ^9^ CNN achieved 95.75% accuracy ^10^ and ANN’s accuracy was 91.08%. Furthermore, we also noticed that SDFL obtains a comparable performance with the benchmark method proposed by the authors of UCI database. ^7^ It is worth nothing that the benchmark method is using multiclass support vector machine for classification; whereas SDFL uses a simpler classifier, i.e. Nearest Neighbour (NN) classifier.

Last but not least, it was discerned that the performance of SDFL is on a par with the Cascade Ensemble Learning model (CELearning). ^16^ Both approaches are ensemble learning methods with multiple layers for data learning. The key difference between these approaches is the analysis algorithms in each layer. CELearning is comprised of four different classifiers, i.e. Random Forest, Extremely Gradient Boosting Trees, Softmax Regression and Extremely Randomized Trees and the final classification result is obtained through the last layer via the score-level fusion of the four complex classifiers. On the other hand, in SDFL, merely Rayleigh coefficient optimization is implemented to extract the discriminant deep features. Further, a simple classifier, i.e. NN classifier, is adopted in SDFL. This deduces that the discrimination capability of SDFL primarily depends on the SDFL modular model to extract discriminant features demanding no complex classifier. From Table 4, we can notice that SDFL just needs ~ $4.3\times 10^{-4}\;$ seconds per sample (sps) for the training phase and ~ $4.2\times 10^{-4}$ sps for the testing phase, on average. The fast feature learning of SDFL and the dimensionality reduction in SDFL to project the data onto a lower-dimensional subspace are the main reasons for having such an efficient computation.

## Conclusions

A cascading learning network for human activity recognition using smartphones is proposed. In this network, a chain of independent discriminant learning modules is aggregated, layer by layer in a stackable framework. Each layer is constituted by a discriminant analysis function and a nonlinear activation function to effectively extract the rich features from the inertial data. SDFL network possesses characteristics of good performance even on small-scale training sample sets, as well as less hyper-parameter fine-tuning, and fast computation compared with the other deep learning networks. Despite showing computational efficiency, the proposed network also demonstrated its classification superiority to most of the state-of-the-art approaches with an accuracy score of ~97% in differentiating human activity classes. In future work, study on small-scale database on SDFL will be investigated.

## Data availability

All data underlying the results are available as part of the article and no additional source data are required.
