# Supplementary material for: Stacked deep analytic model for human activity recognition on a UCI HAR database
Source: F1000Res. 2022 Apr 1;10:1046. Originally published 2021 Oct 15. [Version 3] doi: 10.12688/f1000research.73174.3 (PMC8956851; doi:10.12688/f1000research.73174.3)
Supplement: Peer review history

# Peer review: Reviewer response for version 2

**DOI:** 10.5256/f1000research.121366.r124312

**Published:** 2022-03-25

**Authors:** Cheng-Yaw Low

**Affiliations:** 1 Yonsei University, Seoul, South Korea; 2 Institute for Basic Science, Daejeon, South Korea

The authors have revised the manuscript accordingly. Apart from that, additional contents and experiments have also been included to further demonstrate the performance of SDFL in HAR. The only mandatory correction is the mathematical definition for the total mean vector **μ**, due to two inconsistent variables found >> *N* and *i *should be replaced by *C *and *j*, respectively?

 Since the authors have responded to all my concerns, my final decision is to accept this manuscript without any reservation.

Is the work clearly and accurately presented and does it cite the current literature?

Partly

If applicable, is the statistical analysis and its interpretation appropriate?

Partly

Are all the source data underlying the results available to ensure full reproducibility?

Partly

Is the study design appropriate and is the work technically sound?

Partly

Are the conclusions drawn adequately supported by the results?

Partly

Are sufficient details of methods and analysis provided to allow replication by others?

Partly

Reviewer Expertise:

Stacked neural networks, deep neural networks

I confirm that I have read this submission and believe that I have an appropriate level of expertise to confirm that it is of an acceptable scientific standard.

# Response

**Published:** 2022-03-31

**Authors:** Ying Han Pang

**Affiliations:** Multimedia University, Malaysia

Correction on the mathematical definitions/equations has been performed.

# Peer review: Reviewer response for version 2

**DOI:** 10.5256/f1000research.121366.r124313

**Published:** 2022-03-04

**Authors:** Andrews Samraj

**Affiliations:** 1 Department of Computer Science and Engineering, Mahendra Engineering College, Namakkal, Tamil Nadu, India

I have gone through this revised version, and saw almost all my review points are met by the authors. The introduction and explanation should be elaborated some more so that general readers can understand what real world problem the authors tried to solve. Though the degree of correction is not completely satisfactory, they replied to the points and hence no reservations from my side.

Is the work clearly and accurately presented and does it cite the current literature?

Yes

If applicable, is the statistical analysis and its interpretation appropriate?

I cannot comment. A qualified statistician is required.

Are all the source data underlying the results available to ensure full reproducibility?

Partly

Is the study design appropriate and is the work technically sound?

Yes

Are the conclusions drawn adequately supported by the results?

Yes

Are sufficient details of methods and analysis provided to allow replication by others?

Partly

Reviewer Expertise:

NA

I confirm that I have read this submission and believe that I have an appropriate level of expertise to confirm that it is of an acceptable scientific standard.

# Peer review: Reviewer response for version 1

**DOI:** 10.5256/f1000research.76806.r99185

**Published:** 2021-11-30

**Authors:** Andrews Samraj

**Affiliations:** 1 Department of Computer Science and Engineering, Mahendra Engineering College, Namakkal, Tamil Nadu, India

1. The motivation and contributions may be rewritten to make it simpler to understand the purpose and achievements of the work (purpose of deep features have to be mentioned).
2. Any chances of an Intra personal scatter matrix with any other inter personal scattermatrix?  How was the threshold or borderline decided? There needs to be explanations with sample values.
3. The used hardware and software details, and details about dataset should be shifted from result to any other section in methodology.
4. Slight corrections on technical writings need to be carried out. (eg: Table 4 tabulates: can be changed as either: Table4 presents or it is tabulated in table 4) 
5. A sample subject wise (Personnel) data table would be nice if presented to know the inter personnel differences.

Is the work clearly and accurately presented and does it cite the current literature?

Yes

If applicable, is the statistical analysis and its interpretation appropriate?

I cannot comment. A qualified statistician is required.

Are all the source data underlying the results available to ensure full reproducibility?

Partly

Is the study design appropriate and is the work technically sound?

Yes

Are the conclusions drawn adequately supported by the results?

Yes

Are sufficient details of methods and analysis provided to allow replication by others?

Partly

Reviewer Expertise:

AI, Patterns, Bionics, ML and DEEP Learning, signals,sensors

I confirm that I have read this submission and believe that I have an appropriate level of expertise to confirm that it is of an acceptable scientific standard, however I have significant reservations, as outlined above.

# Response

**Published:** 2022-02-15

**Authors:** Ying Han Pang

**Affiliations:** Multimedia University, Malaysia

First of all, we would like to express our heartiest thanks to the Editor-in-Chief and Reviewers who have provided us with many insightful comments and a chance to improve our works.

1. The motivation and contributions may be rewritten to make it simpler to understand the purpose and achievements of the work (purpose of deep features have to be mentioned).
  Response: The motivation and contribution have been revised for better clarification.
2. Any chances of an Intra personal scatter matrix with any other inter personal scattermatrix?  How was the threshold or borderline decided? There needs to be explanations with sample values.
  Response: The Method section has been revised for better clarification. The optimal projection in SDFL is computed by optimizing the Rayleigh coefficient for modelling the difference between the classes of data through maximizing the ratio of the inter-personal scatter matrix and intra-personal scatter matrix. The variability between data is contained in a subspace spanned by the eigenvectors corresponding to the number of class -1 largest eigenvalues. Hence, the threshold is *C*-1 in this work.
3. The used hardware and software details, and details about dataset should be shifted from result to any other section in methodology.
  Response: The amendment has been done and the details have been shifted to the Method section.
4. Slight corrections on technical writings need to be carried out. (eg: Table 4 tabulates: can be changed as either: Table4 presents or it is tabulated in table 4) 
  Response: The correction has been done.
5. A sample subject wise (Personnel) data table would be nice if presented to know the inter personnel differences.
  Response: Figure 3 is added to illustrate the inter-personnel inertial signal differences of standing activity 

# Peer review: Reviewer response for version 1

**DOI:** 10.5256/f1000research.76806.r97058

**Published:** 2021-11-25

**Authors:** Cheng-Yaw Low

**Affiliations:** 1 Yonsei University, Seoul, South Korea; 2 Institute for Basic Science, Daejeon, South Korea

This manuscript proposes a stacked deep analytic model, dubbed stacked discriminant feature learning (SDFL), for mobile-platform human activity recognition (HAR). Based on the reported experimental results, the layer-wise SDFL trained with small-scale training data outperforms the SOTAs, including the conventional (end-to-end-trained) deep neural networks. 

 My concerns are as follows:

1. Objective 2: The authors claim that SDFL learns low-level to higher-level feature representation. This hypothesis should be demonstrated, if possible. Otherwise, I think this objective should be revised accordingly.
2. Objective 3: The authors should elaborate the subject-independent protocol from the motivation section as a problem statement to be resolved. In addition to that, the advantages of the subject-independent protocol should also be revealed.
3. The methodology section is ambiguous.
  a)  For clarity, the data/feature dimensionalities for all mathematical equations should be indicated accordingly.
  b)  The SDFL hyper-parameters are unknown?
4. The experiment section may not be convincing as it is reliant on only a single dataset, and there is no comprehensive analysis conducted.
  a)  For reproducibility, the parameter configurations should be disclosed.
  b)  It would be better if an ablation study is presented, e.g., exploration of the number of SDFL layers, etc. (refer to [14 ^1^]). Furthermore, the baseline performance, the accuracy for the preprocessed data prior to SDFL learning, is not presented.
  c)  I suggest the authors include additional small-scale HAR datasets for more extensive experiments. 
  d)  I think cross-validation should also be performed.
  e)  I suggest the authors double-check the results reported for precision, recall, f-score, and accuracy. It is rare that these varying matrices give the same value, approximately 96.3%. Alternatively, the confusion matrices for these evaluation matrices should be disclosed.
  (f)  In Table 4, I think the inference time should be computed for the SDFL and other conventional models, in place of the pre-processed data? Otherwise, this comparison may not be meaningful.
  (g) In Table 3, the authors compare SDFL with [24], [25], [4], [3], etc., however, not all are reviewed in the related work section. 

Is the work clearly and accurately presented and does it cite the current literature?

Partly

If applicable, is the statistical analysis and its interpretation appropriate?

Partly

Are all the source data underlying the results available to ensure full reproducibility?

Partly

Is the study design appropriate and is the work technically sound?

Partly

Are the conclusions drawn adequately supported by the results?

Partly

Are sufficient details of methods and analysis provided to allow replication by others?

Partly

Reviewer Expertise:

Stacked neural networks, deep neural networks

I confirm that I have read this submission and believe that I have an appropriate level of expertise to confirm that it is of an acceptable scientific standard, however I have significant reservations, as outlined above.

## References

[1] : Human activity recognition with smartphone sensors using deep learning neural networks. *Expert Systems with Applications* .2016;59: 10.1016/j.eswa.2016.04.032 235-244 10.1016/j.eswa.2016.04.032

# Response

**Published:** 2022-02-15

**Authors:** Ying Han Pang

**Affiliations:** Multimedia University, Malaysia

First of all, we would like to express our heartiest thanks to the Editor-in-Chief and Reviewers who have provided us with many insightful comments and a chance to improve our works.

Objective 2: The authors claim that SDFL learns low-level to higher-level feature representation. This hypothesis should be demonstrated, if possible. Otherwise, I think this objective should be revised accordingly.
  Response: Thanks for the feedback. Authors have revised Objective 2 for better clarification.
 Objective 3: The authors should elaborate the subject-independent protocol from the motivation section as a problem statement to be resolved. In addition to that, the advantages of the subject-independent protocol should also be revealed.
  Response: Authors have included the preference of subject-independent solution in real-time applications in the Motivation section for better relating the motivation and the Objective 3.
a)  For clarity, the data/feature dimensionalities for all mathematical equations should be indicated accordingly. b)  The SDFL hyper-parameters are unknown?
  Response: Authors have included dimensionalities of data/feature, number of training classes *C* and the generated final feature vector in the Methods section for better clarification. Parameters of SDFL are the number of stacking layers, nonlinear activation function, dimensions of intermediate feature vectors and dimensions of the final feature vector. The Method section has been revised to include the information of these parameters for better clarification.
a)  For reproducibility, the parameter configurations should be disclosed. b)  It would be better if an ablation study is presented, e.g., exploration of the number of SDFL layers, etc. (refer to [14 ^1^ ]). Furthermore, the baseline performance, the accuracy for the preprocessed data prior to SDFL learning, is not presented. c)  I suggest the authors include additional small-scale HAR datasets for more extensive experiments. d)  I think cross-validation should also be performed. e)  I suggest the authors double-check the results reported for precision, recall, f-score, and accuracy. It is rare that these varying matrices give the same value, approximately 96.3%. Alternatively, the confusion matrices for these evaluation matrices should be disclosed. (f)  In Table 4, I think the inference time should be computed for the SDFL and other conventional models, in place of the pre-processed data? Otherwise, this comparison may not be meaningful. (g) In Table 3, the authors compare SDFL with [24], [25], [4], [3], etc., however, not all are reviewed in the related work section. 
  Response: The information of these parameters has been included in the Methods section for better clarification. Table 3 has been revised to include the benchmark method, that is Multiclass Support Vector Machine, proposed by the original author of the UCI database. This baseline performance which is the accuracy of the preprocessed data with Support Vector Machine has been included in the table. In order to have a better performance comparison with the existing methods, the testing protocol of this study follows the train-test split protocol defined by the database provider, i.e. this database has a version that is already split into training and test sets that contain data from different participants. This paper is using this version of the database. The results have been double-checked and the confusion matrix of SDFL has been included. Table 4 has been revised by just presenting the computational time of SDFL. With this, readers can have a picture of the training and inference time of the proposed SDFL. The Related Work section has been revised to include the reference in Table 3.
